# Supplementary material for: Effect of prednisolone on glyoxalase 1 in an inbred mouse model of aristolochic acid nephropathy using a proteomics method with fluorogenic derivatization-liquid chromatography-tandem mass spectrometry
Source: PLoS One. 2020 Jan 22;15(1):e0227838. doi: 10.1371/journal.pone.0227838 (PMC6975546; doi:10.1371/journal.pone.0227838)
Supplement: S1 Table — (PDF) [file pone.0227838.s001.pdf]

S1 Table The gradient elution program of FD-HPLC conditions for protein separation

Time (min)	0	10	15	40	60	90	140	150	195	220	420	500	530	560	570
Mobile phase A (%)	94	94	35	30	27	1	1	0	0	0	0	0	0	0	0
Mobile phase B (%)	5	5	30	35	38	44	44	47	48	51	60	70	90	90	100
Mobile phase C (%)	1	1	35	35	35	55	55	53	52	49	40	30	10	10	0
