# Supplementary material for: Effect of prednisolone on glyoxalase 1 in an inbred mouse model of aristolochic acid nephropathy using a proteomics method with fluorogenic derivatization-liquid chromatography-tandem mass spectrometry
Source: PLoS One. 2020 Jan 22;15(1):e0227838. doi: 10.1371/journal.pone.0227838 (PMC6975546; doi:10.1371/journal.pone.0227838)
Supplement: S2 Table — aThe peak numbers correspond to those described in Fig 3. bThe ratio of AA or AA+P groups to N group showed in the table, and the intensity of N group was regarded as “1”. cNCBI processed each consecutive sequence record as GI number, a simple series of digits. N, normal group; AA, aristolochic acid group; AA+P: aristolochic acid+prednisolone. *p < 0.05, significantly different from AA group; # p < 0.05, significantly different from N group. (PDF) [file pone.0227838.s002.pdf]

S2 Table Altered proteins in the kidney homogenate among three groups on Day14

Peak number[a]	N:AA:AA+P (Ratio)	Protein name	Molecular mass (Da)	GI number
**Glycolysis**				
22	1:6.56#:0.87*	Fructose-bisphosphate aldolase B	39,548	gi|15723268
22	1:6.56#:0.87*	Glyoxalase 1	20,826	gi|19354350
22	1:6.56#:0.87*	Pyruvate dehydrogenase E1 α 1	43,204	gi|6679261
28	1:13.40#:4.06	M2-type pyruvate kinase	57,878	gi|1405933
32	1:39.00#:1.85*	Triosephosphate isomerase	26,679	gi|54855
32	1:39.00#:1.85*	Phosphoglycerate kinase	59,716	gi|6679937
32	1:39.00#:1.85*	Aldose reductase	35,725	gi|786001
**Anti-oxidation**				
5	1:0.74:1.28*	Cu/Zn superoxide dismutase	15,752	gi|226471
14	1:0.62#:0.89	Catalase	59,697	gi|442441
18	1:2.22#:0.67*	Glutaredoxin	9,285	gi|31981830
20	1:0.53#:0.82*	Glutathione peroxidase	22,276	gi|2673845
21	1:0.47#:0.80*	Peroxiredoxin V	13,309	gi|7670411
32	1:39.00#:1.85*	Type II peroxiredoxin 1	21,778	gi|3603241
**ATP synthesis**				
18	1:2.22#:0.67*	Ubiquinol-cytochrome c reductase	29,349	gi|13385168
18	1:2.22#:0.67*	Electron transferring flavoprotein, alpha polypeptide	35,018	gi|13097375
18	1:2.22#:0.67*	Cytochrome c oxidase, subunit VIIa 2	9,285	gi|31981830
22	1:6.56#:0.87*	Cytochrome b-5	15,232	gi|13385268
30	1:3.48:0.12*	NADH-ubiquinone oxidoreductase B9 subunit	9,325	gi|21539587
37	1:1.55:0.32*	ATP synthase, H+ transporting, mitochondrial F1 complex, α subunit	59,716	gi|6680748
37	1:1.55:0.32*	Cytochrome b-c1 complex subunit 1	52,735	gi|14548301
**Beta oxidation**				
3	1:0.71#:0.88	Apolipoprotein A-I, isoform CRA b	28,517	gi|148693731
28	1:13.40#:4.06	Medium-chain acyl-CoA dehydrogenase	46,452	gi|6680618

(Cont.)

**Peak number**[a]	**N:AA:AA+P (Ratio)**[b]	**Protein name**	**Molecular mass (Da)**	**GI number**
**Apoptosis**				
14	1:0.62#:0.89	Calreticulin	47,965	gi|6680836
32	1:39.00#:1.85*	Heat shock protein 70 cognate	70,793	gi|309319
**TCA cycle**				
10	1:1.33:1.30*	Aconitase 2	85,410	gi|18079339
37	1:1.55:0.32*	Fumarate hydratase 1	54,336	gi|33859554
37	1:1.55:0.32*	GTP-specific succinyl-CoA synthetase beta subunit	43,830	gi|3766203
**Other**				
3	1:0.71#:0.88	Nicotinamide phosphoribosyltransferase	55,346	gi|10946948
4	1:1.54:0.07*	Y box-binbing protein	35,822	gi|55451
4	1:1.54:0.07*	Nucleolin	76,733	gi|13529464
10	1:1.33:1.30	Cofilin 1	18,548	gi|6680924
10	1:1.33:1.30	AHNAK nucleoprotein isoform 1	603,866	gi|61743961
14	1:0.62#:0.89	alpha-globin	15,132	gi|156257689
19	1:0.52#:0.81	haemoglobin beta-2 chain	16,324	gi|1183933
19	1:0.52#:0.81	S100 calcium binding protein G	8,964	gi|7106263
19	1:0.52#:0.81	Glyceraldehyde-3-phosphate dehydrogenase	35,787	gi|6679937
22	1:6.56#:0.87*	Calmodulin – salmon	16,696	gi|71664
30	1:3.48:0.12*	Tu translation elongation	49,477	gi|27370092
32	1:39.00#:1.85*	Glial fibrillary acidic protein	48,437	gi|51066

[a]The peak numbers correspond to those described in Figure 3. [b]The ratio of AA or AA+P groups to N group showed in the table, and the intensity of N group was regarded as "1". [c]NCBI processed each consecutive sequence record as GI number, a simple series of digits. N, normal group; AA, aristolochic acid group; AA+P: AA+P: aristolochic acid+prednisolone. *$p < 0.05$, significantly different from AA group; # $p < 0.05$, significantly different from N group.
