# Supplementary material for: Effect of prednisolone on glyoxalase 1 in an inbred mouse model of aristolochic acid nephropathy using a proteomics method with fluorogenic derivatization-liquid chromatography-tandem mass spectrometry
Source: PLoS One. 2020 Jan 22;15(1):e0227838. doi: 10.1371/journal.pone.0227838 (PMC6975546; doi:10.1371/journal.pone.0227838)
Supplement: S3 Table — (PDF) [file pone.0227838.s003.pdf]

S3 Table Antibody

Antibody	Catalog no	Company	Clonality	Host	Reactivity
Transforming growth factor-beta 1 (TGF-β) antibody	21898-1-AP	Proteintech Group, Inc	Polyclonal	rabbit	Human, mouse, rat
Matrix metallopeptidase 9	10375-2-AP	Proteintech Group, Inc	Polyclonal	rabbit	Human, mouse, rat, zebrafish, Astragalus membranaceus, hamster, and rabbit
Anti-HGF antibody	ab83760	Abcam	Polyclonal	rabbit	Mouse, Rat, Human
Glyoxalase I antibody	GTX105792	GeneTex	Polyclonal	rabbit	Human, mouse
Beta Actin Antibody	20536–1-AP	Proteintech Group, Inc	Polyclonal	rabbit	Human, mouse, rat, zebrafish, monkey, chicken, goat, Hamster, Orange-spotted groupers, pig, Plateau pika, swine
