# Supplementary material for: Effect of prednisolone on glyoxalase 1 in an inbred mouse model of aristolochic acid nephropathy using a proteomics method with fluorogenic derivatization-liquid chromatography-tandem mass spectrometry
Source: PLoS One. 2020 Jan 22;15(1):e0227838. doi: 10.1371/journal.pone.0227838 (PMC6975546; doi:10.1371/journal.pone.0227838)
Supplement: S1 Fig — The lower, middle, and upper chromatograms were obtained from the kidney homogenates of N- (red), AA- (green), and AA+P- (blue) group mice, respectively. The 47 altered peaks among three groups were numbered. (PDF) [file pone.0227838.s004.pdf]

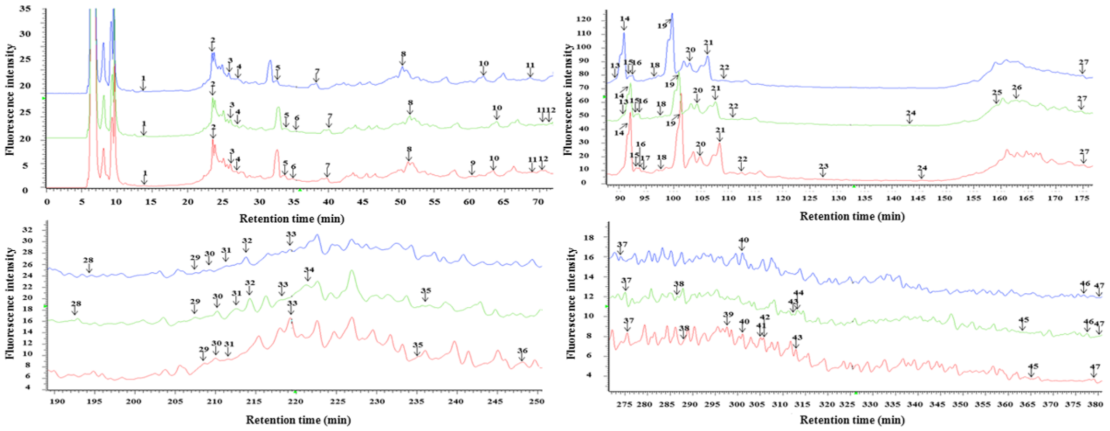

S1 Fig The detailed and amplified chromatograms of Fig 5

The lower, middle, and upper chromatograms were obtained from the kidney homogenates of N- (red), AA- (green), and AA+P- (blue) group mice, respectively. The 47 altered peaks among three groups were numbered.
