# Supplementary material for: Effect of prednisolone on glyoxalase 1 in an inbred mouse model of aristolochic acid nephropathy using a proteomics method with fluorogenic derivatization-liquid chromatography-tandem mass spectrometry
Source: PLoS One. 2020 Jan 22;15(1):e0227838. doi: 10.1371/journal.pone.0227838 (PMC6975546; doi:10.1371/journal.pone.0227838)
Supplement: S2 Fig — AA+P indicated the six chromatograms of AA+P group; AA indicated the six chromatograms of AA group; N indicated the six chromatograms of N group. (PDF) [file pone.0227838.s005.pdf]

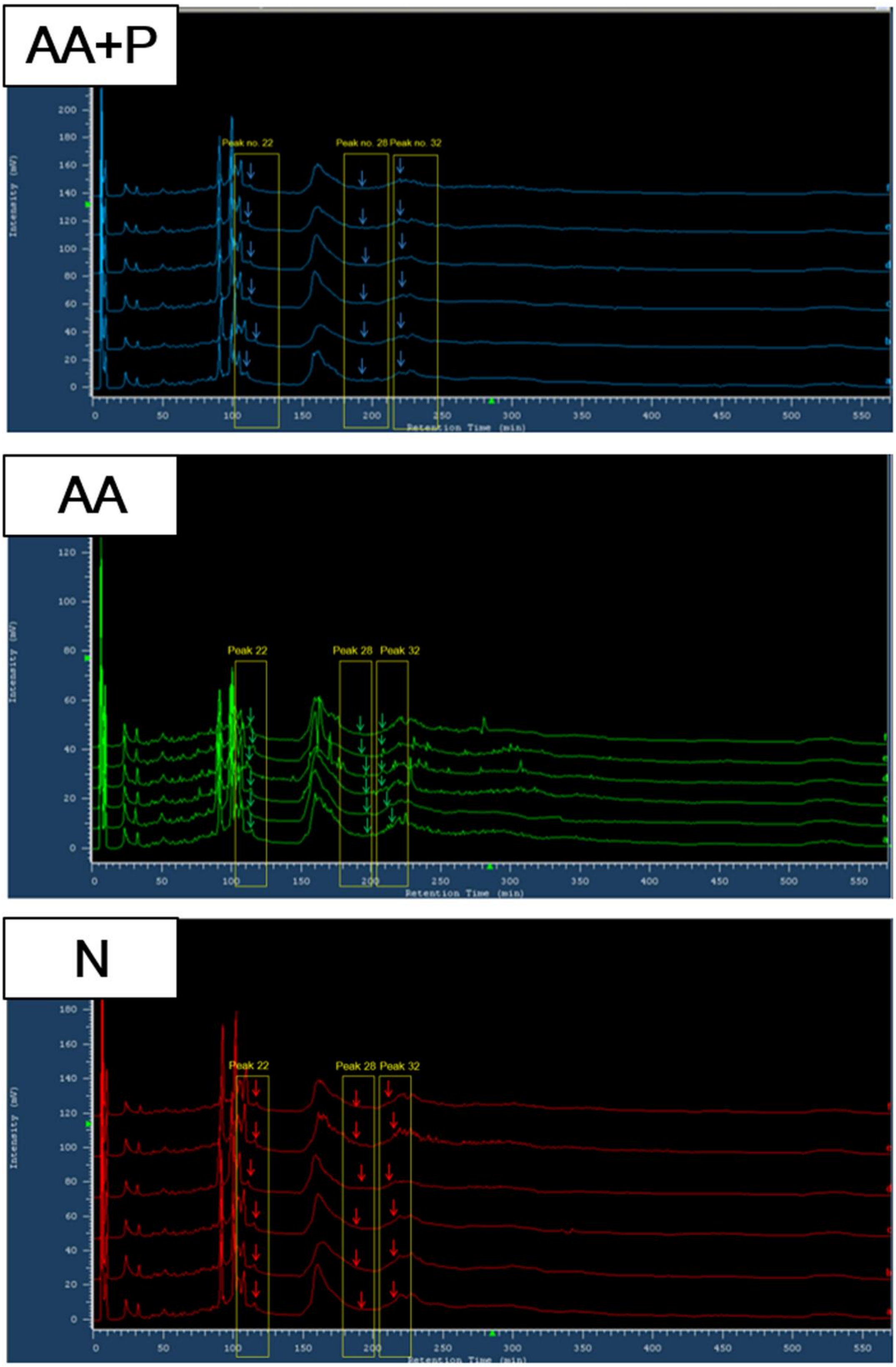

S2 Fig The chromatograms of FD-HPLC for separating proteins.

AA+P indicated the six chromatograms of AA+P group; AA indicated the six chromatograms of AA group; N indicated the six chromatograms of N group.
