# Supplementary material for: Effect of prednisolone on glyoxalase 1 in an inbred mouse model of aristolochic acid nephropathy using a proteomics method with fluorogenic derivatization-liquid chromatography-tandem mass spectrometry
Source: PLoS One. 2020 Jan 22;15(1):e0227838. doi: 10.1371/journal.pone.0227838 (PMC6975546; doi:10.1371/journal.pone.0227838)
Supplement: S3 Fig — (A) Both of the partial membranes which blotted GLO1 and β‐actin antibodies were acquired from the same gel. After the proteins of gel were transferred onto the nitrocellulose membrane, the membrane was blocked with 10% skim milk. Before incubated with primary antibodies, the membrane was cut into two parts. One partial membrane was incubated with anti-GLO1 antibody, and another was incubated with anti-β‐actin antibody, respectively. (B) Both of the band of aldolase B and β‐actin were acquired from the same gel and same membrane. (C) Both of the band of TPI and β‐actin were acquired from the same gel and same membrane. (PDF) [file pone.0227838.s006.pdf]

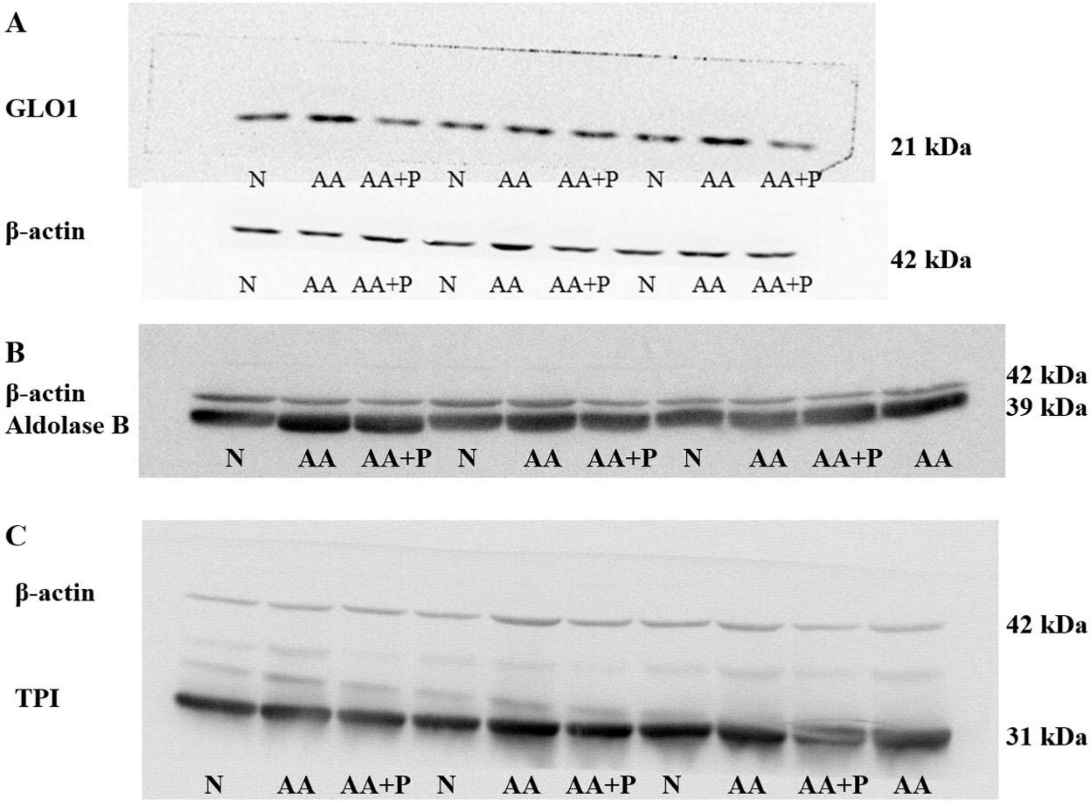

S3 Fig The immunoblotting images for Fig 7

(A)Both of the partial membranes which blotted GLO1 and β-actin antibodies were acquired from the same gel. After the proteins of gel were transferred onto the nitrocellulose membrane, the membrane was blocked with 10% skim milk. Before incubated with primary antibodies, the membrane was cut into two parts. One partial membrane was incubated with anti-GLO1 antibody, and another was incubated with anti-β-actin antibody, respectively. (B) Both of the band of aldolase B and β- actin were acquired from the same gel and same membrane. (C) Both of the band of TPI and β- actin were acquired from the same gel and same membrane.
