# Supplementary figures and images for: Effect of prednisolone on glyoxalase 1 in an inbred mouse model of aristolochic acid nephropathy using a proteomics method with fluorogenic derivatization-liquid chromatography-tandem mass spectrometry
Source: PLoS One. 2020 Jan 22;15(1):e0227838. doi: 10.1371/journal.pone.0227838 (PMC6975546; doi:10.1371/journal.pone.0227838)

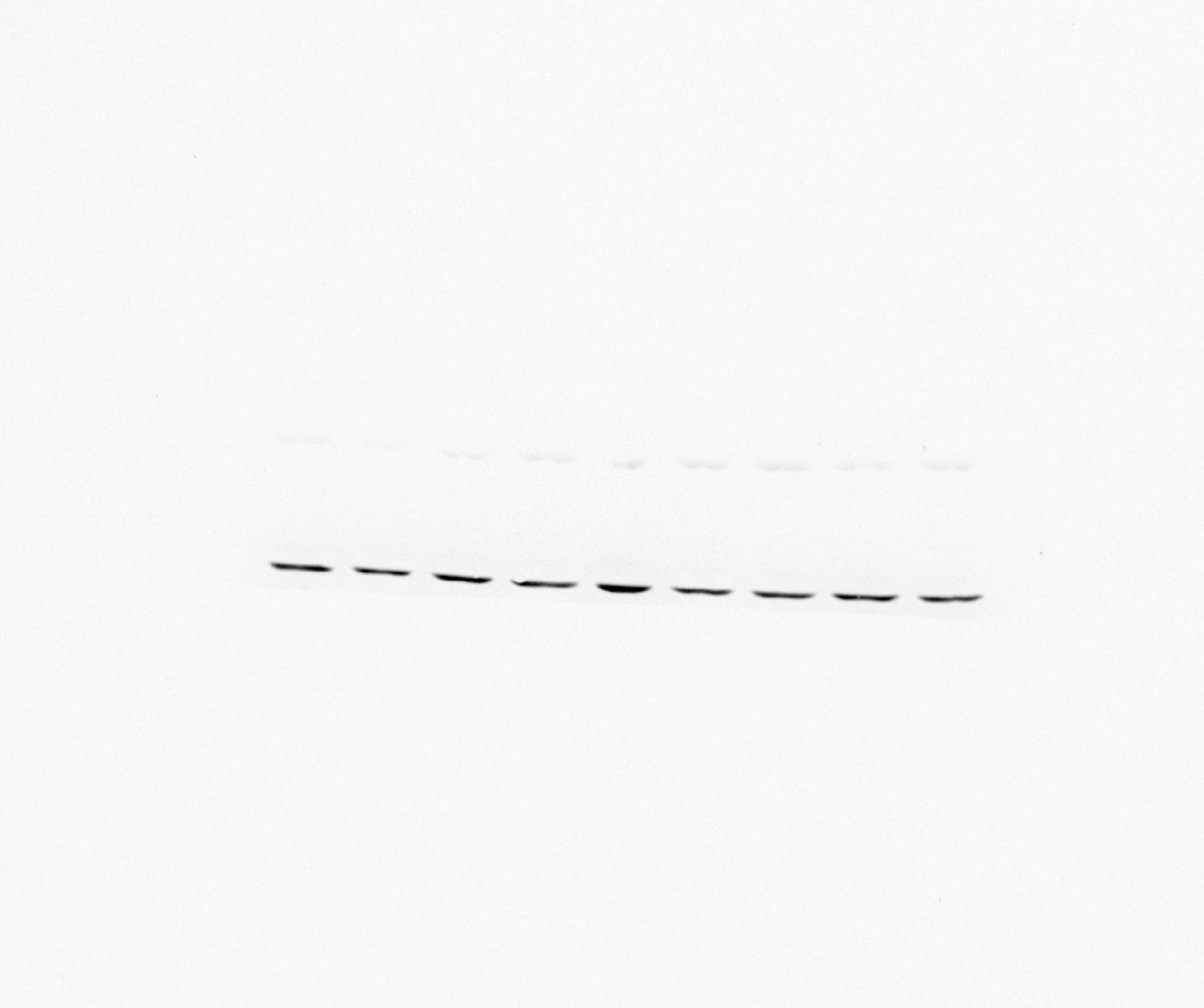

Supplement: S1 Raw Images — (TIF) [file pone.0227838.s007.tif]

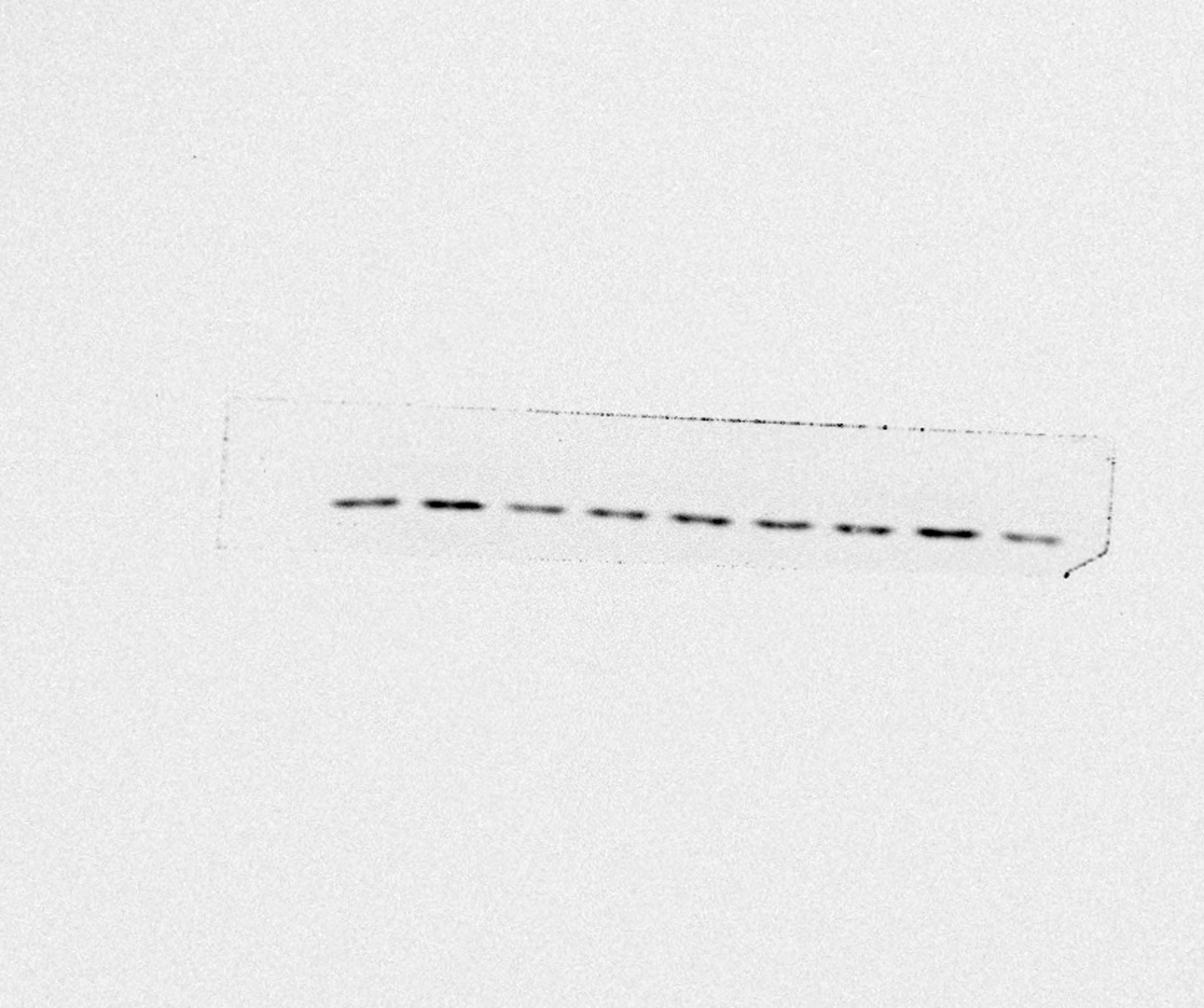

Supplement: S2 Raw Images — (TIF) [file pone.0227838.s008.tif]

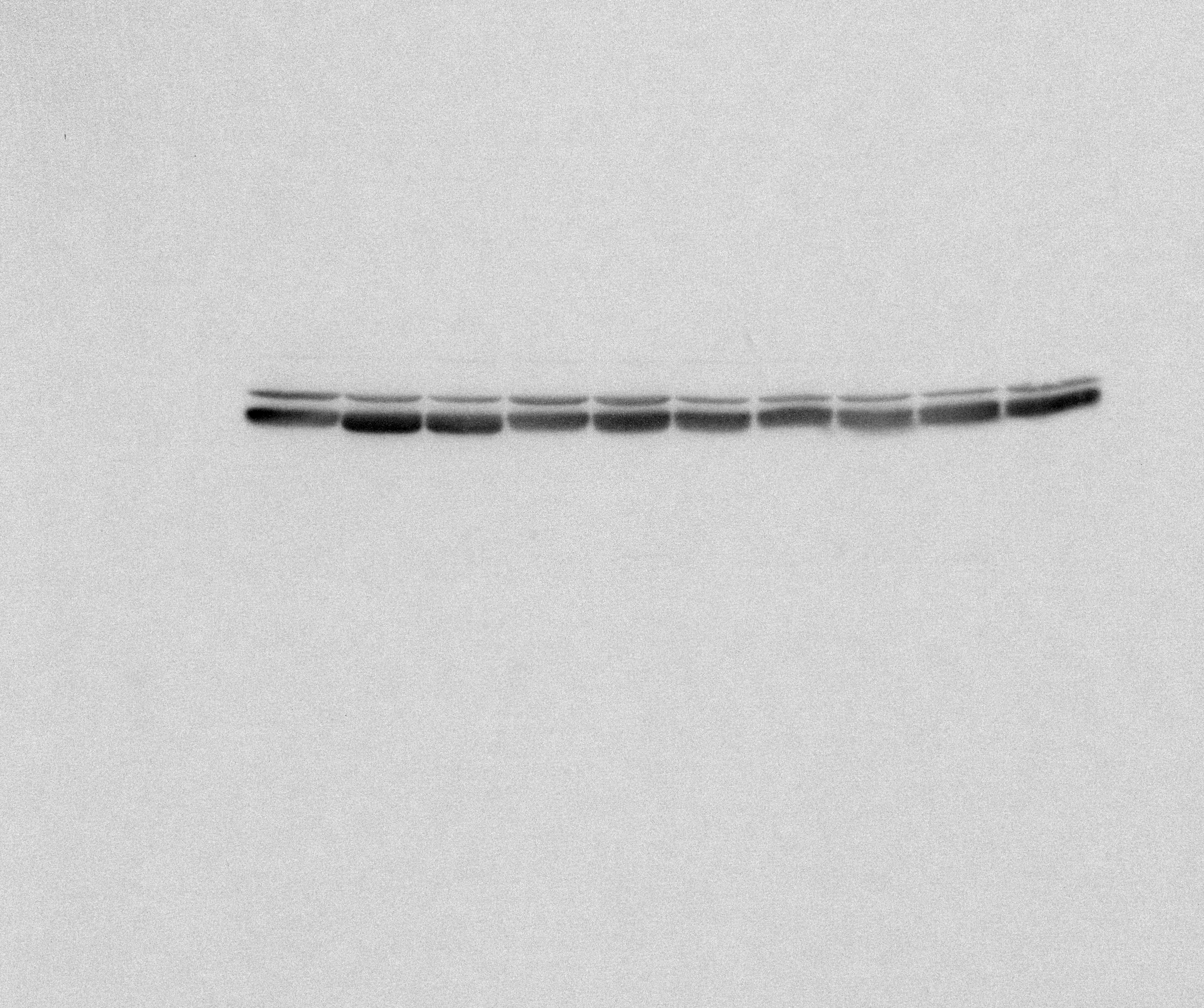

Supplement: S3 Raw Images — (TIF) [file pone.0227838.s009.tif]

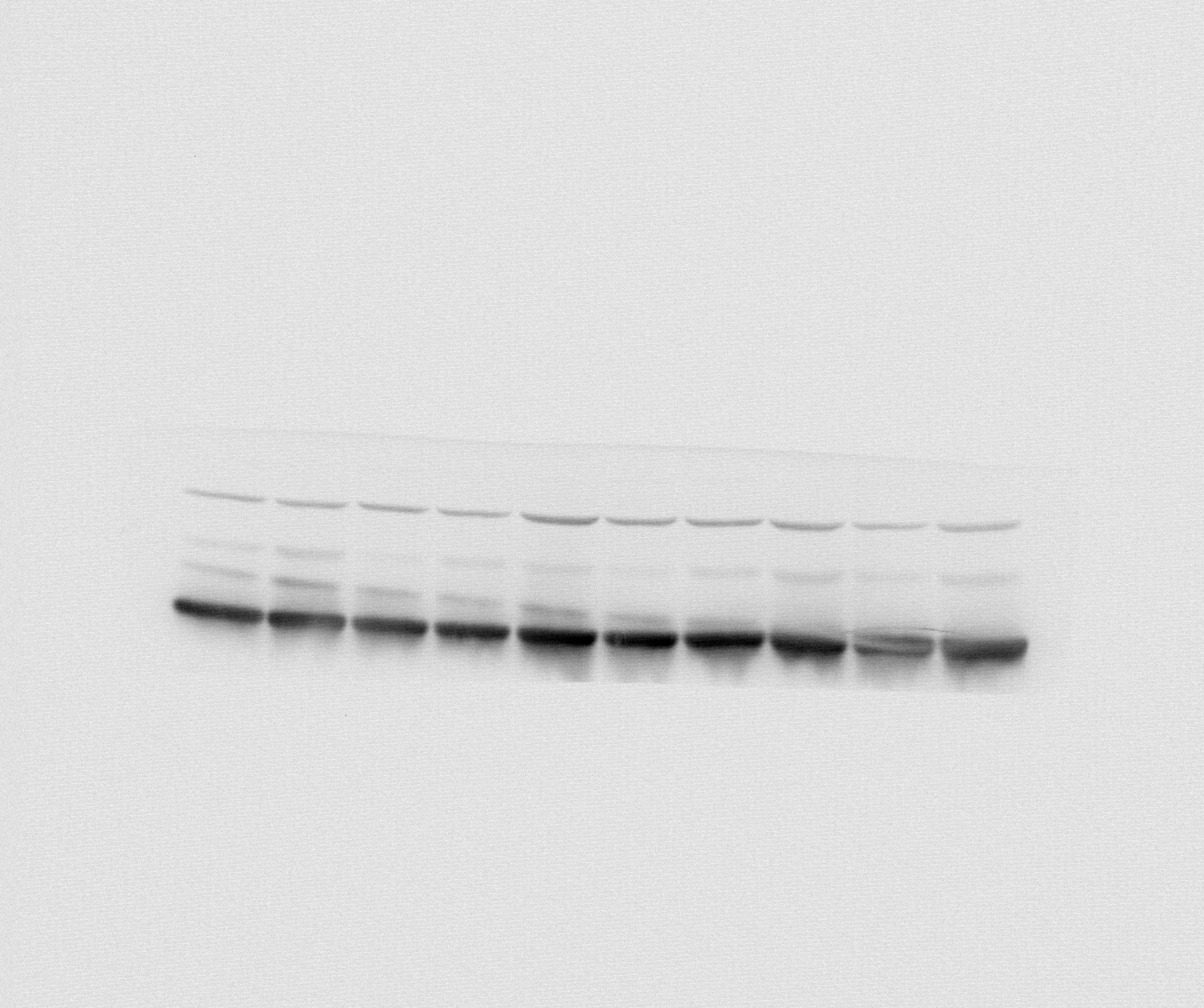

Supplement: S4 Raw Images — (TIF) [file pone.0227838.s010.tif]
