# Supplementary material for: Effect of prednisolone on glyoxalase 1 in an inbred mouse model of aristolochic acid nephropathy using a proteomics method with fluorogenic derivatization-liquid chromatography-tandem mass spectrometry
Source: PLoS One. 2020 Jan 22;15(1):e0227838. doi: 10.1371/journal.pone.0227838 (PMC6975546; doi:10.1371/journal.pone.0227838)
Supplement: Attachment — Submitted filename: Response to reviewer.docx [file pone.0227838.s011.docx]

Dear Reviewers,

Thank you for providing these insights. We have received the letter of 2019/10/27 (PONE-D-19-27780). Our research article was entitled “Effect of prednisolone on glyoxalase 1 in an inbred mouse model of aristolochic acid nephropathy using proteomics method with FD-LC-MS/MS by Shih‐Ming Chen, Chia‐En Lin, Hung-Hsiang Chen, Yu-Fan Cheng, Hui-Wen Cheng, Kazuhiro Imai”. The followings are our response to your suggestion and comment. We highlighted the revised paragraph with **red color** in “Revised Manuscript with Track Changes”, so that you could figure out the the original and revised paragraph.

Reviewer #1:

| Issues 1: The authors may need to consider polishing the language for a better understanding of international researchers. | |
| --- | --- |
| Answer:  We have corrected the English writing again and provided the certificate from the UNIVERSAL LINK CO., LTD. | |
| Issues 2: The authors need to add more discussion and background in the introduction part. | |
| Answer:  We have increase the paragraph to introduce the background and discussion in the part of introduction, especially the mechanisms of aristolochic acid-induced cytotoxicity and carcinogenicity from the recent study. | |
| Original | Revised |
| **Page 4, line 5**  ……The feature of AAN is rapid progression into interstitial renal fibrosis and end-stage renal disease [1, 2]. Despite prohibition of AA-containing herbs, patients are still suffering from AAN [3]. AA injures proximal tubular epithelial cells via the adduction with DNA, which leads to cytotoxicity and carcinogenicity [4]. | **Page 4, line 5**  ……The feature of AAN is rapid progression into interstitial renal fibrosis and end-stage renal disease [1, 2]. AA is extracted from the Aristolochiaceae species and was used for anti-inflammatory activities in traditional medicine. AA is a mixture of 8-methoxy-6-nitrophenanthro-(3,4-D)-1,3-dioxolo-5-carboxylic acid (aristolochic acid I [AAI]) and its 8-demethoxylated form (aristolochic acid II [AAII])[3]. AAI shows stronger nephrotoxicity than AAII in AAN because of the O-methoxy group at position C-8 of the nitrophenanthrene ring [4]. This structure of AAI facilitates AA interactions with DNA, and this AA-DNA adduct leads to cytotoxicity and carcinogenicity. A recent study indicates that AA-induced upper tract urothelial cancer is related to p38 and extracellular signal regulated kinases (ERK) sub-pathways [5]. Despite prohibition of AA-containing herbs, patients are still suffering from AAN [6]. ~~AA injures proximal tubular epithelial cells via the adduction with DNA, which leads to cytotoxicity and carcinogenicity.~~ |
| **Page 5, line 1**  ……Prednisolone fights inflammation via the regulation of tumor necrosis factor-α (TNF-α), interleukin-1 (IL-1), and interleukin-6 (IL-6) etc, thus, widely applied to treating various inflammation diseases or states, such as allergies, asthma, dermatitis, rheumatic disorders, systemic lupus erythematosus, and autoimmune disorders[8-10]. Moreover, prednisolone plays an important role in glucose homeostasis, but the relationship between prednisolone and glycolysis, including the impact of methylglyoxal, a by-product of glycolysis, has never been studied. Therefore, the aim of this study is to (1) explore the effect of prednisolone on changes in glycolysis-related protein expression using the fluorogenic derivatization-liquid chromatography-tandem mass spectrometry (FD-LC-MS/MS) method in the AAN mice model; (2) clarify the pharmacological mechanisms of prednisolone in AA model. | **Page 5, line 10**  ……Prednisolone fights inflammation via the regulation of tumor necrosis factor-α (TNF-α), interleukin-1 (IL-1), and interleukin-6 (IL-6) etc, thus, widely applied to treating various inflammation diseases or states, such as allergies, asthma, dermatitis, rheumatic disorders, systemic lupus erythematosus, and autoimmune disorders[10-12]. Recently, Baudoux *et al.* demonstrated that cluster of differentiation CD4^+^ and CD8^+^ T-cells regulate immune responses in AA-induced acute tubular necrosis [13]. However, the mechanism of prednisolone treatment for AAN is still unclear. Moreover, prednisolone plays an important role in glucose homeostasis, but the relationship between prednisolone and glycolysis, including the impact of methylglyoxal, a by-product of glycolysis, has never been studied. The proteomics study with fluorogenic derivatization-liquid chromatography/tandem mass spectrometry (FD-LC-MS/MS) was introduced in 2004 [14, 15]. This FD-LC-MS/MS method was widely applied to screening proteins in cell lines [16], rat [17], mouse models [18]. Therefore, the aim of this study is was to ~~(1)~~ explore the effect of prednisolone on changes in glycolysis-related protein expression using the fluorogenic derivatization-liquid chromatography-tandem mass spectrometry (FD-LC-MS/MS) method in the AAN mice model~~; (2)~~ and clarify the pharmacological mechanisms of prednisolone in AAN model. |
| Minors : | |
| Please use full name in title line. | |
| Answer:  We have revised our title according to your suggestion, so we have changed our title as the following. | |
| Original | Revised |
| **Title page**  Effect of prednisolone on glyoxalase 1 in an inbred mouse model of aristolochic acid nephropathy using proteomics method with FD-LC-MS/MS | **Title page**  Effect of prednisolone on glyoxalase 1 in an inbred mouse model of aristolochic acid nephropathy using a proteomics method with ~~FD-LC-MS/MS~~ fluorogenic derivatization-liquid chromatography-tandem mass spectrometry |
| Please prepare a figure to illustrate the work-flow for the better understanding. | |
| Answer:  We have prepared a flow chart to explain for our study step by step in Fig 1  **Page 15**    Fig 1 Illustration of flow chart for the current study  Analysis of kidney tissue: (1) The sections of paraffin embedded kidney were used to stained for Periodic Acid-Schiff (PAS) stain and Masson trichrome stain; the cryostat section of kidney were performed immunofluorescence study for transforming growth factor-beta 1 (TGF-β), matrix metallopeptidase 9 (MMP9), and human growth factor (HGF); (2) The kidney homogenate with CHAPS (aq) were used to conduct proteomics study. Before analysis, the proteins were quantitated by BCA protein assay. These proteins of kidney samples were derivatized by DAABD-Cl, and then separated by FD-HPLC. The altered peak fractions were compared and collected which were digested by trypsin, and then these digested fragments were analyzed by LC-MS/MS. Finally, the altered proteins were identified by MASCOT. (3) The immunoblotting analysis for the protein expression for GLO1, TPI, and aldolase B were performed to confirm the findings of proteomics study. Analysis of blood: The blood samples were collected to analyze blood urea nitrogen (BUN) and serum creatinine (Scr). Analysis of urine: The urine samples were collected to analyze activity of urinary NAG, urinary protein, urinary methylglyoxal, and D-lactate.  Aldolase B, fructose-bisphosphate aldolase B; CHAP (aq), 3-[(3-cholamidopropyl) dimethylammonio] propanesulfonic acid(aq); DAABD-Cl, 7-chloro-N-[2-(dimethylamino)ethyl]-2,1,3-benzoxadiazole-4-sulfonamide; FD-HPLC, high performance liquid chromatography with fluorescence detection; GLO1, glyoxalase 1; LC-MS/MS, liquid chromatography-tandem mass spectrometry; TPI, triosephosphate isomerase | |

Reviewer #2: COMMENTS TO AUTHORS
Manuscript Number: PONE-D-19-27780

| (1)As the authors discussed, the fructose 1,6-bisphosphate is cleaved into glyceraldehyde 3-phosphate (GAP) and dihydroxyacetone phosphate (DHAP) catalyzing by aldolase B. To demonstrate that Prednisolone inhibited glycolysis, which consequently led to reduce methylglyoxal, GLO1, and D-lactate the authors should also verify by a conventional immunological assay the increase of the Fructose-bisphosphate aldolase B and TPI. | | |
| --- | --- | --- |
| Answer:  Thanks for your suggestion. We have performed the immunoblotting for aldolase B and TPI to confirm protein expression. Moreover, we have acquired the similar results to the proteomics findings. We have added these new findings in the part of “Method and materials” and “Results”. | | |
| Original | Revised | |
| **Page 10, line12(Method and materials)**  Ten microgram of sample proteins of renal homogenate was loaded into each lane and separated on 12% sodium dodecyl sulfate-polyacrylamide gels using an SDS-PAGE system [11]. The proteins on the gels were transferred onto nitrocellulose membranes. Antibodies against glyoxalase 1 (GLO1; GTX105792, GeneTex, Irvine, CA, USA), β‐actin (20536–1‐AP, Proteintech, Rosemont,IL, USA), and Goat anti-rabbit IgG (H+L), HRP conjugate (SA00001-2, Rosemont,IL, USA) were used at a dilution of 1:1000, 1:2000, and 1:4000, respectively. The signals corresponding to the bands of GLO1 and β‐actin were measured by the TOOL Sensitive ECL kit. ImageJ was used to quantify the intensity of the bands. The relative GLO1 levels were defined as the ratio of GLO1 to β‐actin intensity. | **Page 11, line17(Method and materials)**  Six kidney samples from each group (n=10) were selected to perform immunoblotting analysis. Ten microgram of sample proteins of renal homogenate was loaded into each lane and separated on 12% sodium dodecyl sulfate-polyacrylamide gels using an SDS-PAGE system [18]. The proteins on the gels were transferred onto nitrocellulose membranes. Antibodies against glyoxalase 1 (GLO1; GTX105792, GeneTex, Irvine, CA, USA), β‐actin (20536–1‐AP, Proteintech, Rosemont,IL, USA), fructose-bisphosphate aldolase B (aldolase B, GTX101363, GeneTex, Irvine, CA, USA), triosephosphate isomerase (TPI, GTX104618, Irvine, CA, USA), and Goat anti-rabbit IgG (H+L), HRP conjugate (SA00001-2, Rosemont,IL, USA) were used at a dilution of 1:1000, 1:2000, 1:3000, 1:3000, and 1:4000, respectively. | |
| **Page 24, line2 (Results)**  On day 14, the GLO1 protein expression in the kidney homogenate of the N (100.0 ± 30.9%) and AA+P (108.9 ± 10.7%) groups were significantly lower than those of the AA group (191.60 ± 61.1%) (Fig 6), which were similar to the FD‐LC-MS/MS proteomics findings. | | **Page 30, line2 (Results)**  The differences in GLO1, aldolase B, and TPI expressions were similar among the three groups (Fig 7A, B, C). On day 14, the GLO1 protein expression in the kidney homogenate of the N (100.0 ± 30.9%) and AA+P (108.9 ± 10.7%) groups were significantly lower than those of the AA group (191.60 ± 61.1%) (p<0.05) (Fig 67D), which were similar to the FD‐LC-MS/MS proteomics findings. Moreover, aldolase B protein expression in the kidney homogenate of the N (100 ± 36.3%) and AA+P (136.2 ± 54.7%) groups were significantly lower than those of the AA group (223.6 ± 52.5%) (p<0.05) as shown in Fig 7E.TPI protein expression in the kidney homogenate of the N (100.0 ± 19.6%) and AA+P (121.4 ± 9.3%) groups were significantly lower than those of the AA group (181.5 ±25.9%) (p<0.05) (Fig 7F).  All the immunoblotting were shown in S3 Fig. |
| **Page 25, line 1 (Figure 6 legend)**  Figure 6 Immunoblotting analysis of GLO1 in kidney homogenates on day 14.  Expression of GLO1 and β‐actin among the three groups on day 14 (A). The β‐actin was used as the internal standard. (B) Semi-quantitation of the relative GLO1 level. The expression of relative GLO1 level decreased in the AA+P group compared with the AA group. N, normal group; AA, aristolochic acid group; AA+P: AA+P: aristolochic acid+prednisolone; GLO1, glyoxalase 1. * *p* < 0.05 significantly different from the AA group; # *p* < 0.05 significantly different from the N group. | | **Page 31, line (Figure 7 legend)**  Figure ~~6~~7 Immunoblotting analysis of GLO1, aldolase B, and TPI in kidney homogenates on day 14.  ~~Expression of GLO1 and β‐actin among the three groups on day 14 (A).~~ Expression of GLO1 (A), aldolase B(B), and TPI (C) among the three groups on day 14. The β‐actin was used as the internal standard. ~~(B)~~ (D) Semi-quantitation of the relative GLO1 level. (E) Semi-quantitation of the relative aldolase B level. (F) Semi-quantitation of the relative TPI level. The expression of relative GLO1, aldolase B, and TPI level decreased in the AA+P group compared with the AA group. N, normal group; AA, aristolochic acid group; AA+P: AA+P: aristolochic acid+prednisolone; GLO1, glyoxalase 1~~.~~; aldolase B, fructose-bisphosphate aldolase B;TPI, triosephosphate isomerase. * *p* < 0.05 significantly different from the AA group; # *p* < 0.05 significantly different from the N group. |
| (2) Its not clear along all the paper how many samples have been used for the different experiments (i.e, how many samples were used for WB analysis? | | |
| Answer:  All mice of biochemical data (including BUN, Scr, urinary protein, and NAG), sections (PAS stain, Masson trichrome stain, and immunofluourance), urinary methylglyoxal, and urinary D-lactate were determined. We selected six mice from each group to perform proteomics study. We selected six mice from each group to perform immunoblotting for glyoxalase1, aldolase B, and triphosphate isomerase because of the rest of the kidney tissue. | | |
| Original | Revised | |
| Page 6, line6  **Biochemical assays** | Page 7, line 3  **Biochemical assays**  All biochemical parameters from each of the 30 mice were measured. | |
| (No describing) | Page 7, line11  **Histological examination**  All of the kidneys from the 30 mice were embedded with paraffin, and the sections were sliced into 4 to 5 µm sections [18, 19]. The kidney sections were stainedwith periodic acid–Schiff (PAS) (395B, Sigma-Aldrich, Inc., MO, USA) and Masson trichrome stain (HT15, Sigma-Aldrich, Inc., MO, USA) according to the manufacturers’ instructions. | |
| Page 6, line14  **Immunofluorescence study**  Immunofluorescence study was performed as previous experiment [11]. | Page 7, line18  **Immunofluorescence study**  Samples from kidneys from the 30 mice were prepared for ~~I~~immunofluorescence study which was were performed previously ~~as previous experiment~~ [18]. | |
| Page 7, line10  **Proteomics study**  **Sample preparation** | Page 8, line16  **Proteomics study**  **Sample preparation**  Six kidney samples from each group (n=10) were selected for the proteomics study. | |
| Page 10, line11  **Immunoblotting analysis** | Page 11, line17  **Immunoblotting analysis**  Six kidney samples from each group (n=10) were selected to perform immunoblotting analysis. | |
| Page 11, line 3  **Determination of urinary methylglyoxal** | Page 12, line 12  **Determination of urinary amount of methylglyoxal**  Urinary methylglyoxal amount from all of the mice were determined. | |
| Page 11, line 14  **Preparation of urine sample** | Page 13, line 6  **Preparation of urine sample**  Urinary D-lactate amount from all of the mice were determined. | |
| (3) Figure 4  I think , each group chromatogram should be reported with its proper Fluorescence intensity, otherwise chromatograms comparison is not possible. | | |
| Answer:  We have reported the chromatograms according to your suggestion. The chromatograms with the correct scales were shown in Fig 5. | | |
| **Original** | | **Revised** |
|  | |  |
| 4) Figure 5  Why did you use peak height instead of peak area to calculate the differences among the 3 different groups of samples? From S1_Fig , peak n. 22 seems higher in Normal (red line) instead of AA (green line); peak n. 28 seems not to be the same in the 3 different chromatograms as well as for peak n.32, which seems to be higher in Normal instead of AA. | | |
| Answer:  Usually, peak area is better than peak height. In our case, each peak has different baseline. If we calculated with peak area, the intensity of the peak might include part of other peak area. This FD-LC-MS/MS method used peak height rather than peak area according to the previous study (Ichibangase et al., Journal of Proteome Research; 2007, 6, 2841-2849).  About this question “From S1_Fig , peak n. 22 seems higher in Normal (red line) instead of AA (green line); peak n. 28 seems not to be the same in the 3 different chromatograms as well as for peak n.32, which seems to be higher in Normal instead of AA.”, we have labeled peak no.22, 28, and 32 of each chromatogram of sample in each group in S2_Fig. According to the results of S2_Fig, we acquired the intensity and expressed as mean ± SD in Table 2 and Table S2. All of this intensity is not only acquired from S1_Fig. Thus, we average the intensity of six sample data from each group and compared among three groups. | | |
| 5) Figure 8  From Figure 8 A it doesn’t seem that the amount of urinary total lactate in the N group was lower than that in the AA and AA+P groups, instead it seems that the amount of urinary total lactate in the N group was higher than that in the AA and AA+P groups. Please clarify. | | |
| Answer:  Answer: Figure 8 A and 8C indicated the **“level”** of total lactate according to the chromatograms but not the **“amount”** of total lactate. Figure 8B and 8D indicated the **“level”** of D-lactate according to the chromatograms but not the **“amount”** of D-lactate. The amount of total lactate was expressed as: amount of lactate (mmol) = level of lactate (mM) x urinary volume (µL); Expression of D-lactate was similar to expression of total lactate (amount of D-lactate [µmol] = level of lactate [µM] x urinary volume [µL]). Because the level of lactate is very low, we use the amount which could more reflect how much methyglyoxal metabolize into D-lactate in mouse body. | | |
| **Original** | | **Revised** |
| **Page 11, line 3**  **Determination of urinary methylglyoxal**  ……The amount of urinary methylglyoxal was defined as level of methylglyoxal × urinary volume. | | **Page 12, line 11**  **Determination of urinary amount of methylglyoxal**  Urinary methylglyoxal amount from all of the mice were determined. ……The amount of urinary methylglyoxal was defined as level of methylglyoxal × 12 h-urinary volume. |
| **Page 11, line 14**  Determination of urinary D-lactate | | **Page 13, line 4**  Determination of urinary amount of D-lactate |
| **Page 12, line 10**  ……The flow rate was set 0.7 mL/min for 0–35 min and 0.9 mL/min for 35.1–60 min [12, 21]. | | **Page 14, line 3**  ……The flow rate was set 0.7 mL/min for 0–35 min and 0.9 mL/min for 35.1–60 min [19, 27]. The amount of urinary lactate was defined as lactate levels ×12 h urinary volume. |
| **Page 12, line 12**  **Enantio-separation of D-lactate**  ……The derivatives were detected at an emission wavelength of 547 nm with an excitation of 491 nm [12, 21]. | | **Page 14, line 4**  **~~Enantio-separation~~ Enantiomeric separation of D-lactate**  ……The derivatives were detected at an emission wavelength of 547 nm with an excitation of 491 nm [19, 27]. Urinary D-lactate amount were defined as levels of D-lactate ×12 h urinary volume. |
| 6) Table 1  Missing biochemical data at basal (T0) could clarify the differences of the animal groups at the beginning of the study, useful to appreciate and confirm the effect of therap | | |
| Answer: We have determined the urinary data (urinary protein and NAG) on baseline according to your suggestion. However, it is not impossible to acquire the blood data on baseline, because it is necessary to acquire more than 60 µL of blood sample so that we could analyze the data. The body weight of mouse is about 20 g, and it is a high stress to get such volume of blood for a mice. We only can get such large volume, when the mouse were sacrificed. In order to solve this problem, we compared blood data of the “**normal group”** with AA and AA+P groups on day 14. | | |
| Original | | Revised |
| **Page 13, line 8**  **Biochemical assays**  BUN (20.67 ± 0.73 vs. 23.00 ± 2.09 mg/dL, p < 0.05), Scr (0.27 ± 0.04 vs. 0.36 ± 0.06 mg/dL, p < 0.05), NAG (2.22 ± 0.42 vs. 2.94 ± 0.13 U/L, p < 0.05), and urinary protein (1.75 ± 0.68 vs. 2.94 ± 0.09 mg/day, p < 0.05) in the AA+P group were significant lower in the AA group on day 14 (Table 1). | | **Page 17, line 3**  **Biochemical assays**  There were no significant differences at baseline in NAG (N [1.93 ± 0.15 U/L] vs. AA [1.92 ± 0.12 U/L] vs. AA+P [1.95 ± 0.10 U/L]) and urinary protein (N [1.21 ± 0.34 mg/day] vs. AA [1.17 ± 0.29 mg/day] vs. AA+P [1.26 ± 0.34 mg/day]) among the three groups. BUN (20.67 ± 0.73 vs. 23.00 ± 2.09 mg/dL, p < 0.05), Scr (0.27 ± 0.04 vs. 0.36 ± 0.06 mg/dL, p < 0.05), NAG (2.22 ± 0.42 vs. 2.94 ± 0.13 U/L, p < 0.05), and urinary protein (1.75 ± 0.68 vs. 2.94 ± 0.09 mg/day, p < 0.05) in the AA+P group were significant lower in the AA group on day 14 (Table 1). |
| Original | | |
| **Page 14, line 1** **Table 1. Biochemical data of the N, AA, and AA+P groups on day 14** Group \| BUN (mg/dL) \| Scr (mg/dL) \| NAG (μM/min/L) \| UP (mg/day); N \| 17.85 ± 1.91** \| 0.22 ± 0.07** \| 1.93 ± 0.06** \| 1.26 ± 0.20**; AA \| 23.00 ± 2.09 \| 0.36 ± 0.06 \| 2.94 ± 0.13 \| 2.94 ± 0.09; AA+P \| 20.67 ± 0.73* \| 0.27 ± 0.04* \| 2.22 ± 0.42* \| 1.75 ± 0.68* The BUN, Scr, NAG activity, and UP excretion of N and AA+P group mice significantly decreased on day 14 compared with those of AA-group mice. N, normal group; AA, aristolochic acid group; AA+P: AA+P: aristolochic acid + prednisolone; NAG, N-acetyl-β-D-glucosamine; BUN, blood urea nitrogen; Scr, serum creatinine; UP, urinary protein. *p < 0.05, ** p < 0.01 significantly different from the AA group | | |
| Revised | | |
| **Page 19, line1** Table 1. Biochemical parameters of the normal (N), aristocholochic acid (AA), and aristocholochic acid + prednisolone (AA+P) groups on day 14 Group \| NAG (μM/min/L) \| \| UP (mg/day) \| \| BUN (mg/dL) \| Scr (mg/dL); \| Baseline \| Day 14 \| Baseline \| Day 14 \| Day 14 \| Day 14; N \| 1.93 ± 0.15 \| 1.93 ± 0.06** \| 1.21 ± 0.34 \| 1.26 ± 0.20** \| 17.85 ± 1.91** \| 0.22 ± 0.07**; AA \| 1.92 ± 0.12 \| 2.94 ± 0.13 \| 1.17 ± 0.29 \| 2.94 ± 0.09 \| 23.00 ± 2.09 \| 0.36 ± 0.06; AA+P \| 1.95 ± 0.10 \| 2.22 ± 0.42* \| 1.26 ± 0.34 \| 1.75 ± 0.68* \| 20.67 ± 0.73* \| 0.27 ± 0.04* There were no significant difference of NAG and UP among N, AA, and AA+P groups at baseline. The BUN, Scr, NAG activity, and UP excretion of N and AA+P group mice significantly decreased on day 14 compared with those of AA-group mice. Baseline was defined as the day at the beginning of experiment (before the mice were administrated AA). N, normal group; AA, aristolochic acid group; AA+P: AA+P: aristolochic acid + prednisolone; NAG, *N*-acetyl-β-D-glucosamine; BUN, blood urea nitrogen; Scr, serum creatinine; UP, urinary protein. **p* < 0.05, ** *p* < 0.01 significantly different from the AA group. | | |

I appreciated very much that reviewers gave us a practical suggestion and comment to our work. Thank you very much!

Sincerely yours,

Shih-Ming Chen, Ph.D.

School of Pharmacy, College of Pharmacy,

Taipei Medical University,

No. 250, Wuxing St., Taipei 11031, Taiwan

Tel: 886-2-2736-1661 ext. 6176

Fax: 886-2-2736-6518

E-mail: smchen@tmu.edu.tw
